# Donor Selection for Allogenic Hemopoietic Stem Cell Transplantation: Clinical and Ethical Considerations

**DOI:** 10.1155/2017/5250790

**Published:** 2017-06-07

**Authors:** Irene Riezzo, Natascha Pascale, Raffaele La Russa, Arcangelo Liso, Monica Salerno, Emanuela Turillazzi

**Affiliations:** ^1^Institute of Legal Medicine, Department of Clinical and Experimental Medicine, University of Foggia, Ospedale “Colonnello D'Avanzo”, Viale Degli Aviatori, 1, 71122 Foggia, Italy; ^2^Istituto Clinico-Scientifico Malzoni, 83100 Avellino, Italy; ^3^Department of Anatomical, Histological, Forensic and Orthopaedic Sciences, Sapienza University of Rome, Viale Regina Elena 336, 00185 Rome, Italy; ^4^Institute of Hematology, Department of Medical and Surgical Sciences, University of Foggia, Viale Pinto 1, 71122 Foggia, Italy

## Abstract

Allogenic hematopoietic progenitor cell transplantation (allo-HSCT) is an established treatment for many diseases. Stem cells may be obtained from different sources: mobilized peripheral blood stem cells, bone marrow, and umbilical cord blood. The progress in transplantation procedures, the establishment of experienced transplant centres, and the creation of unrelated adult donor registries and cord blood banks gave those without an human leucocyte antigen- (HLA-) identical sibling donor the opportunity to find a donor and cord blood units worldwide. HSCT imposes operative cautions so that the entire donation/transplantation procedure is safe for both donors and recipients; it carries with it significant clinical, moral, and ethical concerns, mostly when donors are minors. The following points have been stressed: the donation should be excluded when excessive risks for the donor are reasonable, donors must receive an accurate information regarding eventual adverse events and health burden for the donors themselves, a valid consent is required, and the recipient's risks must be outweighed by the expected benefits. The issue of conflict of interest, when the same physician has the responsibility for both donor selection and recipient care, is highlighted as well as the need of an adequate insurance protection for all the parties involved.

## 1. Introduction

Allogenic hematopoietic progenitor cell transplantation (allo-HSCT) is an established therapeutic strategy for many hematologic malignancies, bone marrow failure syndromes, and metabolic diseases [1]. In allo-HSCT, a relatively small inoculum of donor hematopoietic cells is called upon to recapitulate a diverse and fully functional hematopoietic system in the recipient [2].

Stem cells for HSCT may be obtained from different sources: mobilized peripheral blood stem cells (PBSCs), bone marrow (BM), and umbilical cord blood (UCB) [3].

The progress in transplantation procedures, the establishment of experienced transplant centres, and the creation of unrelated adult donor registries and cord blood banks gave those without an HLA-identical sibling donor the opportunity of a concrete hope to find a donor and cord blood units worldwide [4].

Overall, the donation of organs and tissues represents a fundamental resource for all humanity, an example of altruism with the aim of helping sick people in need. If we tend to relate to the beneficial effect of donation, we can say that donation may represent a sort of ethical duty for all people; on the other hand, health institutions have a perfectly symmetrical duty to guarantee safety for donors. Careful selection of potential donors, clinical evaluation and management of the entire donation/transplantation procedure by experts, and a proper communicative process represent the key points of HSCT [5].

The transplantation procedure is complex both from a clinical and from an ethical point of view. Ethical and clinical concerns related to the different phases of the process (pretransplantation care, donor search, harvest, transplantation phase, and short and long-term follow-up) are likely to overlap and intersect each other [6].

## 2. The Beginning

After the detonation of nuclear weapons in the Second World War, there was a surge in knowledge of the biology of hematopoiesis and the possible use of HSCT as a rescue strategy for radiation-induced bone marrow injury [7].

The first studies performed in mice led to remarkable results in hematopoiesis and suggested insights regarding potential clinical use. The first steps toward understanding the mechanisms of hematopoietic stem arise from the observation made by Jacobson and colleagues that in mice treated with lethal irradiation, the spleen protection allowed the conservation of hematopoiesis [8]. Is due to Lorenz et al. the first evidence about the infusion of bone marrow in mice, as hemopoietic recovery after radiation injury [9]. In 1957, Thomas and colleagues lead the first experiment on human beings, with the treatment of acute leukaemia with a bone marrow infusion from fetal and adult cadavers [10]; importantly, the authors reported the observation of an immunological reaction mediated by the graft against the leukaemia cells.

For several years, the research on murine and canine models fed the comprehension that transplantation of bone marrow leads to hematopoietic recovery after injury, both heterologous and autologous infusion [11, 12] and that the infusion is related to immune reaction mediated by genetic factors, nowadays known as graft-versus-host disease (GVHD) [13, 14].

The subsequent steps of those studies led to an understanding of the crucial role of the histocompatibility antigens, up to the discovery of the human leukocyte antigen (HLA) that codes for the major histocompatibility complex (MHC) [15–19].

In the early seventies, the first allogenic transplants of bone marrow were limited to congenital and acquired bone marrow failure syndromes, immunodeficiencies, and advanced refractory leukaemia [20–22]. By 1980, the curative potential of HSCTs had encouraged its use in malignancies previously considered “incurable,” such as chronic myelogenous leukaemia. Allo-HSCT was also increasingly utilized as a curative therapy not only for severe aplastic anaemia but also for other severe nonmalignant conditions, such as thalassemia, sickle cell anaemia, and inborn metabolism errors as well [7].

A historical step in the strategy of HSCTs has been the collection of a stem cell product through venous access rather than through a bone marrow harvest. The discovery of the physiological presence of CD34 hematopoietic stem cells in the peripheral blood and the administration of mobilizing cytokines allows the greater expansion of the number of stem cells [23–26].

In 1989, Gianni et al. utilized the granulocyte colony-stimulating factor (G-CSF) to mobilize bone marrow stem cells into the peripheral blood, thus favouring the collection of peripheral blood stem cells [27], used both for autologous and for allo-HSCT [28].

The choice of bone marrow or peripheral blood as source for allogeneic transplantation in adults is still under study and evaluation [29].

In 1988, for the first time, a HLA-matched sibling successfully donated cord blood-derived stem cells to a patient affected by Fanconi anaemia [30].

## 3. The Clinical Challenge

Historically, HLA-matched donors are the most suitable for allo-HSCT. However, progress in studies allows to state that it is not necessary [31].

This is especially true when it is considered the significant expansion of the donors thanks to the introduction of the Reduced Intensity Regimens [22].

The Reduced Intensity Regimens was developed over a period of several years. Santos and colleagues introduced a combination of busulfan and cyclophosphamide [32], followed by several methods of reduced conditioning regimens available in human transplants [33].

The reduced regimen often produces mixed repertoire from both donor and recipient cells that after transplant, it turns in a conversion to a donor-derived hematopoietic cell and T-cell population. These approaches overcome the historical problem of age limit for HSCT [7]. However, relapse is still a critical point in these cases and myeloablative conditioning remains standard in younger patients with rapidly proliferating malignancies [34]. It has been reported that some ethnic groups have an increased susceptibility to the immune-mediated adverse drug reactions (ADRs), a phenomenon related to HLA allele frequency [35].

The possibility of allo-HSCT from unrelated donors is definitely the result of the increased knowledge of the HLA system and the improved HLA typing techniques. The registries of unrelated donors show a remarkable ethnical variability, for example, the odds of finding a full match for a Caucasian patient is approximately 70%, whereas this number is 18% for African Americans. One or two antigen-mismatched transplants have been carried out in this situation, but the incidence of graft failure and GVHD remains problematic [7]. Every HLA antigen mismatch between the donor and the recipient has been shown to adversely affect the success of allogeneic transplant [36].

The donor age does not appear as a limit for the allo-HSCT. It is proved that it does not determine the risk of prolonged neutropenia or thrombocytopenia of delayed engraftment and graft rejection and of development of malignant clonal disorder. Furthermore, no major long-term adverse effects on GVHD and 5-year nonrelapse mortality have been seen with grafts from older donors. It is noticed also that the transplant from older sibling donors is associated with lower risk of acute GVHD grades II to IV compared with younger donors [2].

Cord blood and partially HLA-matched related (haploidentical) donors represent a new approach for allo-HSCT that overcome HLA barriers and the related limit due to high rates of graft rejection and GVHD. These modern approaches to donor selection mean that for any patient who need transplant, there is a possible donor. Anyway, the critical problems related to transplant procedures, such as risk of GVHD, relapses, infectious complications, remain a challenge to overcome.

UCB transplantation is suitable also when MHC-mismatched donors are enrolled, because the risk of GVHD is lower than other transplantations [37]. The T-cell repertoire is entirely established at birth, as well as the CB B-cell repertoire [38–41]. The clonal predominance in complex T-cell populations (polyclonal versus oligoclonal or monoclonal profiles), detected by the analysis of complementary-determining regions (CDR) length, is the major difference between the adult and the CB T-cell repertoire. The absence of antigenic exposure can be related to the peculiar polyclonal profiles observed in CB T-cell. This condition could explain the lower rate of GVHD reported after CB transplantation [42].

## 4. Donor Selection

In BM donation, drug administration is not required. Hemopoietic stem cells are aspirated from the hips, which requires general or regional anaesthesia and a brief hospitalization for the donor [4, 43]. The main risks are those related to anaesthesia, but postdonation deaths have been described, due to cardiorespiratory arrest, pulmonary embolism, sickle cell crisis, and stroke [44]. Although the incidence of severe and fatal adverse events is very rare [4], severe blood loss, wound infection, and pain at the site of puncture have been reported as potential morbidity risks [45]. Fatigue, low back pain, headaches, nausea, walking difficulties, and sleep disorders represent the most frequent short-term negative events related to bone marrow donation. Rarely, long-term adverse effects may occur, such as chronic pain at the donation site and the need of iron supplementation and, even, of blood transfusion in smaller children who donate for larger recipients [46–49].

The repeated injections of G-CSF in periferal blood (PB) transplantation may be associated with bone pain. Overall, due to the procedure of PB donation, risks of heterologous blood products, thrombocytopenia, and spleen rupture have been described [50–52].

In 2001, the World Marrow Donor Association (WMDA) established a reporting registry for serious adverse events due to stem cell donation by unrelated donors [4]. The most frequent adverse events in BM donation were cardiac arrest and those related to anaesthesia [53]. BM donors most often reported pain at the collection site and anaesthesia site; fatigue was the frequently reported symptom [54].

Six serious adverse reactions (as defined by the WMDA) were reported in those donors making a subsequent donation (either for the same patient or a different patient, *n* = 107), a rate of 5.6%. Although this rate of serious adverse reactions differed statistically from that in first-time donors (RR = 3.29, *P* = 0.005), overall numbers were small [55].

Over the time, the WMDA has guaranteed uniformity, quality, and safety of unrelated donations (UDs); on the contrary, critical questions remain for related donors (RDs). Situation of conflicts of interest (a member of the medical team engaged in both donation and transplantation), the lack of standardized clinical eligibility criteria and of centralization of adverse event, and donor follow-up reporting system are challenging points [56].

In 2010, the Ethics Working Group and the Clinical Working Group of the WMDA issued recommendations for family donor care [57]. In 2013, the Worldwide Network for Blood and Marrow Transplantation (WBMT) issued a consensus statement on the standardization of the assessment of donor outcome [58].

Some evidence from the literature suggest that hematopoietic progenitor cell (HPC) donation is safer for UDs than RDs. Halter et al. reported the early death (<30 days) of five related donors in 36,317 family donations between 1993 and 2005 while no deaths were reported in 14,706 donations from volunteer UDs [53].

Other authors reported similar findings [59], mostly focusing on the lack of adherence to the clinical eligibility criteria [60, 61].

An Italian survey reported that only 26.4% donors underwent thorough screening in accordance with Italian Bone Marrow Donor Registry standards [62].

Standard certified criteria and terms for medical suitability of RDs should be guaranteed [57].

In family donations, psychological as well as physical well-being has to be specifically addressed. Apart from physical, clinical effects and consequences of the donation procedure and psychological impact of donation may be significant both in positive and in negative sense. Undoubtedly, the act of donation can be experienced in a positive way by the donor; in the case of negative outcome of the transplant, psychological difficulties may arise for the donor, particularly if his/her recipient dies or develops severe adverse events [63, 64].

The need of a strict donor follow-up has been recently highlighted, focusing on the reporting of any serious adverse reaction occurring early (<30 days) after the donation procedure. Furthermore, a long-term follow-up of HPC donors is desirable once every one-two years for at least 10 years [58, 65, 66].

The issue of safety is reported as critical with particular attention to the comparison between allogeneic BM and PBSC donations [67]. Both BM and PB donation procedures may be burdened by the same psychologic effects, fatigue, and reduced energy after the procedure. More severe pain at the donation site, greater incidence of hemorrhage, anaemia, and hypotension, and a tendency to have more days of restricted activity and hospitalization may occur related to BM donation. Even if a greater number of adverse events in the BM group (56%) compared with those in the PB group (44%) are reported, there is no clear evidence of which collection method is safer for the donor [46, 67–71].

UCB has been used successfully since 1988 as a source of hematopoietic stem cells for transplantation involving sibling donors and, more recently, unrelated recipients [72, 73]. UCB donation may represent a less critical procedure from the point of view of HLA matching, because lymphocytes in cord blood have a minor immunological reactivity than lymphocytes from older donors [74].

Due to the widespread use of UCB donation, public cord blood banking becomes a reality, where units donated by anybody can be used by anybody else [73]. Scientific societies made a strong case for public cord blood banking, and national and international efforts aimed at expanding collection and storage [75–77], as when the number of units stored in public banks increases, the chances of finding a matching unit also increase.

Public cord banks coexist with private cord blood registries that counsel the storage of a newborn infant's cord blood in the unlikely event that siblings suffer from a disease necessitating a transplant [78]. The chances of self-use when stored in private banks are slim [79]. In general, public banking is favoured by the American Academy of Pediatrics, the American Medical Association, the American College of Obstetrics and Gynaecology, and the American Society for Blood and Marrow Transplantation [80–82].

Unrelated UCB is an alternative source of allo-HSCT with a less stringent need to match HLA types as compared with harvesting HSC from bone marrow or nonneonatal peripheral blood. However, failure to restore hematopoiesis after allogeneic UCB transplantation due to HLA-specific antibodies in the recipient and the small number of cells from UCB undermines the potential for therapeutic success [83–85].

## 5. The Issue of Children

Children may be a source of hematopoietic stem cell most commonly in the case of children requiring transplant. Potentially, children may also donate for adults (sibling, parent, or other family members) [86, 87].

Two thousand allo-HSCT are performed annually in children in the USA, many on research protocols. For approximately one-third, the HSC donor is also a child, typically a healthy sibling who undergoes bone marrow harvest or large volume apheresis for collection of peripheral blood stem cells [1].

Because donation of HC does not improve the donor's own physical health and carries a risk of side effects, careful assessment of medical risks specific to the individual donor must be considered, as well as the ethical and legal aspects associated with donation from a child [88].

Generally, the risks related to donation by minors are modest and rarely severe adverse events are reported [46, 51, 89]. Particularly in the paediatric donor population, a long-term follow-up is substantial and it is even more necessary if children are treated with G-CSF. There is no direct medical benefit from serving as a stem cell donor. The benefit is always stated as the psychosocial benefit of helping a sibling or other close family members. However, the donation procedure may carry medical risks, mostly related to anaesthesia and more rarely to nerve, bone, or tissue injury [47, 90–92].

From an ethical point of view, the central knot in stem cell donation by a minor is carefully considering and balancing the risks and benefits of the potential donor and, on the other hand, the contextual perils and advantages to the recipient and to his or her family [93, 94].

Recognizing that HSC donors face risks without the potential for direct medical benefit, the American Academy of Pediatrics (AAP) recently published guidelines specifying when minors may ethically serve as HSC donors for a standard (nonresearch) transplant [51, 92, 95]. In the 2011 statement of AAP, five criteria were proposed to be fulfilled so that the donation by a minor could be ethically acceptable [86].

The absence of a medically equivalent histocompatible adult relative suitable for donation is the first imperative criterion. Within this conceptual paradigm, several subsequent steps are to be followed. In the case of multiple histocompatible, clinically equivalent siblings, the age of the potential donor above or closest to that of consent represents a priority criterion. The AAP statement takes note of the complexity of a parental decision-making process in stem cell donation by a minor and of the difficulties of the eventual sequential search for an unrelated donor through the international registries that may be time consuming, frustrating, unsuccessful, and very expensive [93]. Therefore, the simultaneous, not sequential, search of stem cell research from all the potential sources appears ethically acceptable. Secondly, the AAP underlines the ethical and moral problematic nature of asking a minor to donate for an unknown, emotionally distant relative. Consequently, it is opportune that an interpersonal healthy relationship between the donor and the recipient exists. Donation for strangers from minors and their enrollment in international registries are unacceptable except for cord blood donations.

The likelihood that the recipient will benefit from transplantation represents the third criterion. The donor's psychosocial burden of a disastrous transplant has to be carefully weighted when a donation by a minor is taken into consideration [96], particularly when the potential recipient is a sibling.

This issue is strictly intertwined with the fourth condition that reinforces the need of a careful balance between the overall risks to the donor and the benefits expected both to the donor and to the recipient. In the light of this criterion, the proper selection of the method of stem cells collection is pivotal. It is noteworthy that the inclusion of the potential child donors in the decision-making process and other strategies as medical play acting may be useful in minimizing psychological and emotional burden related to the donation procedure [97–101].

Finally, it is mandatory that parental permission and donor assent are obtained.

Conclusively, medical criteria for donor selection, independent assessment, and a donor follow-up are substantial in the paediatric setting as in the adult one. Some peculiar issues related to the paediatric population, such as the role of the minors in parental decisional process, and the relationship between the donor and the potential recipients within the familial context deserve particular attention [47, 90].

## 6. Informed Consent: How Much Information?

The legal and ethical framework within which we are moving is the assumption that the human subject's voluntary consent to any kind of medical treatment is absolutely essential. This means that the person involved should have the capacity to give consent, should be in such a position as to be able to exercise free power of informed choice, without any form of constraint or coercion, and should have enough knowledge and comprehension of the issue involved in his/her condition as to enable him/her to make an understanding and enlightened decision. For decision makers to make an informed choice, especially about tissue, organ, and blood donation, they must have a firm understanding of the processes, risks, and benefits associated with donation.

Several elements should be considered critical with regard to informed consent to tissue and blood donation. The first of these is the conceptual paradigm that in stem cells donation, regardless of the sources and as in all types of donation, there is no medical benefit to the donor. Every kind of donation is of vital interest to society as a whole. However, the traditional physician-patient relationship is affected by the fact that the patient is the subject of donation rather than the subject of treatment. Donation does not purport to benefit the subject; instead, the subject helps the potential recipient of his/her donation. Donors consent to the “invasion” of their person or to procedures, the results of which are of potential benefit to others (the recipients). Thus, the difficulty of quantitatively and qualitatively measuring the extent of the risks related to donation has to be counterbalanced with the measurement of the benefits for future, potential recipients. Accepting this concept requires acceptance of the subsequent concept that it is necessary to balance the potential benefit to the recipients with the potential risks to the donors. The degree of risks for the donor should never be excessive. When this balance between risks and benefits is achievable, the following step is the respect for the donor's autonomy, as realized through their informed consent.

In this regard, a fundamental issue is the amount of information which is to be provided to the donor. How much is to be told about the donation procedure and how should it be told? What risks of donation should be explained? Are some risks to be concealed from the patient?

Different types of HSC sources imply different types of risks related to donation. The ways in which HSC are obtained, as well as the way and type of information provided to donors about benefits and risks, is likely to influence a person's willingness to donate. While there is no doubt that the donor's voluntary and informed consent is the ethical and legal prerequisite for donation, it may be debatable, from an ethical point of view, how much information is adequate and how much choice donors should have.

One of the major ethical issue in the context of donation is therefore the amount of the information. In fact, if consent must be informed, it is fundamental that the information should be truthful and complete. But, in this peculiar context, the problematic dimension of risk disclosure is perceived. In fact, it is not a matter of simply disclosing the risks of the donation but also taking into account other elements related to the donor, that is, to the recipient of the informative message, such as the donor's real desire and determination to donate, and his/her psychological condition and level of knowledge. Donors' interpretation of and responses to uncertainty may depend on their personal characteristics and values and may be affected by the manner in which uncertainty and risks related to donation are communicated [102–104] and reported that when initially agreeing to donate, donors had hardly any information about the procedure and its associated risks. This suggests that the decision was not the result of a logical process but rather an emotional reaction. The reactions were towards a terminally ill recipient, a fear of loss, the desire to save a loved one, or an outgrowth of family, social, or moral expectations. The results of studies investigating the accession process to living kidney donation were similar [105–107]. The same concerns may arise also in hematopietic stem cells donation. A greater focus on risk perception by donors is thus needed since, without it, real and full freedom of choice is unlikely.

Even more thorny concerns arise when the proposed donor is a minor. When children do serve as living donors, these donations raise serious ethical issues that mainly focus on an even more careful benefit/burden balance in the perspective of avoiding any possible risk to the paediatric donor.

## 7. Conflict of Interest

It seems opportune to dedicate a number of reflections to the question of the potential conflict of interest, which may arise in allo-HSCT practice.

The phenomenology of the possible conflicts of interest is vast and highly polymorphous, the very concept of “conflict of interest” not always being clear and univocal [108]. Broadly speaking, there is a conflict of interest when professional judgement concerning a primary interest (the health of a patient, for example) tends to be unduly influenced by a secondary interest (economic gain, personal advantage) with conscious violations of obligations. More subtle situations where good judgement may be affected unknowingly exist [108].

It appears evident that in some cases of medical practice, the doctor-patient relationship may be influenced by the figure of a third person. In these cases, conflicts of interest are more likely.

Such is the case of allo-HSC transplantation in which there are two patients: the donor (who, in general, has no medical benefits from the donation) and the potential recipient (for whom the procedure may be lifesaving). The issue is even more complex when the donors and the recipients are relatives, often siblings [109].

When a single physician has the responsibility for both donor selection and recipient care, his/her sound judgement may be unknowingly affected. In donation-transplantation relationships, undue involuntary influences on the physician may occur and the interest of one party (recipient or donor) may be perceived by the physician to be stronger. It appears inevitable that the judgement of the physician who manage the donor could be “vitiated” by the influence of the suffering, the illness, and the impending death of the recipient. For this reason, both the National Marrow Donor Program (NMDP) and the WMDA [110] state that the medical evaluation of the unrelated donor must be performed by a physician who is not a member of the transplantation team caring for the patient.

In real practice, however, medical management of recipients and their related donors by the same provider is common [109–111]. In 2012, a survey of Italian transplant centres reported a high percentage of cases in which donors and recipients were cared by the same member of the medical team [62]. Similar results have been found in other recent surveys carried out both in Europe [111] and in the USA [109] even though the recommended practice is that the donor and recipient are assessed by different experts.

## 8. Liability and Insurance

HSCT may be considered a single phenomenon which, from the standpoint of medical malpractice, may show two faces of the same coin.

The first concerns potential malpractice claims related to adverse, undesirable events in the recipients. These may involve malpractice actions by recipients who may have developed serious complications (such as life-threatening infections) or death following the transplantation. Furthermore, clinical failure may be alleged, since, despite the fact that HSC transplantation can be curative for many disorders and malignancies yet for many conditions, success rates remain frustratingly low and morbidity and mortality remain unacceptably high. Physicians are required to follow a known pattern of therapy and treatment on a sick individual. Treatment is explained to the patient (or to his/her legal guardian) before it is administered, and the patient agrees (or not), knowing (as far as possible) the predictable anticipated results as well as the possible complications. Liability is imposed and remedies set for failure. In the case of litigation, courts are called to judge the physicians responsible for the cure of the transplant recipients following the established principles and legal requirements of medical liability [112]: a breach of duty on the part of the physicians to provide adequate care and treatment to the patient, a deviation from the standards of the profession, a causal relationship between such breach of duty and injury to the patient, and the existence of damages that flow from the injury. The remedy of insurance schemes is intended to provide compensation when injury or death results from lack of skill or negligence and, finally, in accordance with national legislations, even from an inadequacy of informed consent.

The second issue concerns malpractice claim alleged by hematopoietic stem cells donors. It is noteworthy that it has been reported that the majority of the donors of bone marrow and peripheral blood stem cells are satisfied with their donation and complain only of transient minor side effects. Only few donors suffer more severely and/or for more protracted negative events. Serious complications and side effects, or death, due to bone marrow or peripheral blood stem cell donation are extremely rare [113]. As in the case of stem cells recipients, potential malpractice allegations may be related to any adverse events or physical harm (for example, due to drug administration in peripheral blood stem cells donors and to anaesthesia in bone marrow donors) caused by any of the events of the donation chain, including those prior to donation. Furthermore, because of the peculiarity of the act of donation, the issue of autonomy and consent by donors may assume even greater importance in malpractice and doctors' negligence in failing to fully explain to donors that the risks involved with the donation at the time of consent may attract claim allegations.

Since the primary responsibility of donor registries and transplantation centres is to protect the donors and ensure their safety, in the case of any adverse event related to any phase of the donation process, the registry or the centre should offer benefits to all stem cell donors. These benefits might be provided through insurance coverage [114]. Moving from the first guidelines issued by WMDA in 1994 [115], the assumption that life and disability insurance should be obtained for each donor before the harvest procedure is indispensable. In the latest guidelines [116], the general rule expressly dealing with insurance remedy is strongly reaffirmed: “The registry must assume responsibility and establish procedures for all donor medical expenses including the pre-collection physical examination, the collection procedure and all post-collection medical expenses that are directly related to the donation. No volunteer donor should assume financial liability for any portion of the follow up testing and/or HPC procurement process. The registry is responsible for all reasonable expenses incurred by the donor. The registry, or its designee, should offer disability and death benefits to all volunteer donors. The registry should maintain liability insurance.”

As part of the communication process, donors have to be informed of the existing liability insurance scheme of the registry or centre, focusing on its content and limitations. Any limitation in insurance must be clearly explained to the donor [113].

Finally, since the international exchange of stem cells and the percentage of products crossing international borders are more and more increasing [117], international standards regarding the coverage and level of compensation of insurance are mandatory [113].

## 9. Conclusions

HSCT is a modern day success story, an irreplaceable therapeutic opportunity, and a factual relevance unimaginable until some years ago. For recipients, both adults and children, HSCT may represent the only plausible therapeutic solution. For the donor and their relatives, something good, especially from a psychological point of view, may emerge from donation. For the medical profession and for the society as a whole, there is an opportunity to bring about a cure for otherwise intractable diseases. However, even on the awareness of the deep significance and therapeutic importance of HSCT, the challenges are great. HSCT imposes operative cautions so that the entire donation/transplantation procedure is safe for both donors and recipients. Furthermore, HSCT carries with it significant clinical, moral, and ethical concerns, mostly when donors are minor and the donation process occurs within the familial context. The saviour sibling issue is a clear example of the potential complexity and psychological burden of decision-making process in this peculiar context.

In several diseases, HSCT may be the best solution in terms of survival and quality of life; however, it is not without problems. The ethical and juridical importance of consent cannot be neglected. The act of donation is likely to be dictated by altruism or great solidarity; the concern is that, in some situations (i.e., when donors are minor and the donation act is within the family context), situations that could force on donors (i.e. the possibility of psychological pressure on the donor) can occur, even if at an unconscious level. The conceptual paradigm of consent lies on the protection of donors' autonomy and personal dignity. National and international efforts must aim at avoiding any kind of coercion, commercialization, and economic gain in the act of donation.

Finally, HSCT may present significant conflict of interest to medical staff, which may be less or more perceived when a single physician has the responsibility for both donor selection and recipient care. The roller coaster of emotions involved in these situations can overwhelm the critical, clinical judgement of the physician.

In light of the previous discussion, the following points should be stressed: the donation should be excluded when excessive risks for the donor are reasonable; donors must receive an accurate information regarding eventual adverse events and health burden for the donors themselves; a valid, free, and informed consent, to prove the freedom of an altruistic act like the donation, is required; and the recipient's risks must be outweighed by the expected benefits. Finally, there is a strict need of an adequate insurance protection for all the parties involved in allo-HSCT procedures.
